# Spontaneous breathing trial and post-extubation work of breathing in morbidly obese critically ill patients

**DOI:** 10.1186/s13054-016-1457-4

**Published:** 2016-10-27

**Authors:** Martin Mahul, Boris Jung, Fabrice Galia, Nicolas Molinari, Audrey de Jong, Yannaël Coisel, Rosanna Vaschetto, Stefan Matecki, Gérald Chanques, Laurent Brochard, Samir Jaber

**Affiliations:** 1 Intensive Care Unit, Anaesthesia and Critical Care Department, Saint Eloi Teaching Hospital, Centre Hospitalier Universitaire Montpellier, 80 avenue Augustin Fliche, F-34295 Montpellier, Cedex 5 France; 2 Department of Statistics, University of Montpellier Lapeyronie Hospital, UMR 729 MISTEA, Montpellier, France; 3 Anaesthesia and Intensive Care Medicine, Maggiore della Carità Hospital, Novara, Italy; 4 Centre National de la Recherche Scientifique (CNRS 9214) - Institut National de la Santé et de la Recherche Médicale (INSERM U-1046), Montpellier University, Montpellier, France; 5 Keenan Research Centre, St Michael’s Hospital, Toronto, Canada; 6 Interdepartmental Division of Critical Care Medicine, University of Toronto, Toronto, Canada

**Keywords:** Weaning, Mechanical ventilation, Obese, Work of breathing, Acute respiratory failure

## Abstract

**Background:**

Predicting whether an obese critically ill patient can be successfully extubated may be specially challenging. Several weaning tests have been described but no physiological study has evaluated the weaning test that would best reflect the post-extubation inspiratory effort.

**Methods:**

This was a physiological randomized crossover study in a medical and surgical single-center Intensive Care Unit, in patients with body mass index (BMI) >35 kg/m^2^ who were mechanically ventilated for more than 24 h and underwent a weaning test. After randomization, 17 patients were explored using five settings : pressure support ventilation (PSV) 7 and positive end-expiratory pressure (PEEP) 7 cmH2O; PSV 0 and PEEP 7cmH2O; PSV 7 and PEEP 0 cmH2O; PSV 0 and PEEP 0 cmH2O; and a T piece, and after extubation. To further minimize interaction between each setting, a period of baseline ventilation was performed between each step of the study. We hypothesized that the post-extubation work of breathing (WOB) would be similar to the T-tube WOB.

**Results:**

Respiratory variables and esophageal and gastric pressure were recorded. Inspiratory muscle effort was calculated as the esophageal and trans-diaphragmatic pressure time products and WOB. Sixteen obese patients (BMI 44 kg/m^2^ ± 8) were included and successfully extubated. Post-extubation inspiratory effort, calculated by WOB, was 1.56 J/L ± 0.50, not statistically different from the T piece (1.57 J/L ± 0.56) or PSV 0 and PEEP 0 cmH_2_O (1.58 J/L ± 0.57), whatever the index of inspiratory effort. The three tests that maintained pressure support statistically underestimated post-extubation inspiratory effort (WOB 0.69 J/L ± 0.31, 1.15 J/L ± 0.39 and 1.09 J/L ± 0.49, respectively, *p* < 0.001). Respiratory mechanics and arterial blood gases did not differ between the five tests and the post-extubation condition.

**Conclusions:**

In obese patients, inspiratory effort measured during weaning tests with either a T-piece or a PSV 0 and PEEP 0 was not different to post-extubation inspiratory effort. In contrast, weaning tests with positive pressure overestimated post-extubation inspiratory effort.

**Trial registration:**

Clinical trial.gov (reference NCT01616901), 2012, June 4th

**Electronic supplementary material:**

The online version of this article (doi:10.1186/s13054-016-1457-4) contains supplementary material, which is available to authorized users.

## Background

Extubation is a critical decision in the Intensive Care Unit (ICU). Extubation failure may occur in up to 20 % [1] of patients and is associated with morbidity. Excessive and non-sustainable work of breathing (WOB) is likely a major reason for extubation failure [2–5]. Evaluation of how the critically ill patient is breathing with no assistance or a minimal level of assistance (the period known as the weaning test or the spontaneous breathing trial) [4] is therefore recommended before extubation [3, 4, 6, 7]. Different weaning tests are suggested for non-selected adult patients: a T-piece trial (oxygen supply without positive pressure), continuous positive airway pressure (CPAP) and low pressure support ventilation (PSV), with a low level of PSV, from 5 to 8 cmH_2_O, to compensate for the imposed workload due to the ventilator circuit [3, 4, 6, 7]. Although these weaning tests are not equivalent in term of the WOB [8, 9] and studies are underpowered to assess the risk of extubation failure, they are recommended to assess whether a patient is ready to be extubated [3, 6].

Predicting whether an obese critically ill patient can be successfully extubated may be specially challenging. Obesity decreases respiratory system compliance, inspiratory and expiratory lung volumes, functional residual capacity, upper airway mechanical function and neuromuscular strength [10]. Moreover, in obese patients, oxygen consumption is increased, with a high proportion of this consumption spent in the WOB [11–13]. Although the T piece, CPAP and low PSV levels have been used to reproduce post-extubation conditions in non-selected critically ill patients, the weaning test modality that would best reproduce post-extubation inspiratory effort (WOB and pressure time product indexes) in obese critically ill patients has never been evaluated and many clinicians are worried about using no support during the test [14, 15].

The aim of our study was thus to assess which weaning test would best reproduce post-extubation inspiratory effort in obese critically ill patients. We compared a T-piece trial to weaning tests with PSV 7 and positive end-expiratory pressure (PEEP) 7 cmH_2_O; PSV 0 and PEEP 7 cmH_2_O; PSV 7 and PEEP 0 cmH_2_O; PSV 0 and PEEP 0 cmH_2_O, in this particular population. We hypothesized that the T-tube or PSV 0 and PEEP 0 cmH_2_O would best approximate the post-extubation WOB.

## Methods

### Study

This was a physiological prospective randomized crossover study (Additional file 1: Table S1), approved by the Ethics Committee of the Saint-Eloi Teaching Hospital (2012 A-00294-39, Comité de Protection des Personnes Sud Méditerranée III, Montpellier, France), and registered on clinical trial.gov (reference NCT01616901, registered June 4th, 2012). All patients provided their written informed consent.

### Patients

Upon admission, height and weight were measured using the bed scale and a tape measure. All morbidly obese patients, defined by a body mass index (total body weight in kg/height in m^2^) >35 kg/m^2^ [16], were considered eligible for inclusion in the study if they were mechanically ventilated for at least 24 h and were considered by the physician on duty to be ready for extubation. Patients were not included in the study if there was any contraindication to the insertion of an esophageal catheter.

### Experimental procedure and study design

**Fig. 1:**
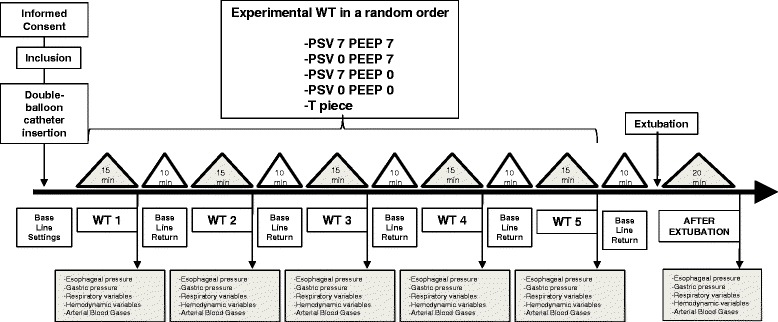
Study design. Eleven morbidly obese patients ventilated in pressure support ventilation (*PSV*) and positive end-expiratory pressure (*PEEP*), considered as baseline settings, were included to randomly perform the five weaning test modalities of the study before extubation: PSV 7 cmH_2_O + PEEP 7 cm H_2_O; PSV 0 cmH_2_O + PEEP 7 cmH_2_O; PSV 7 cmH_2_O + PEEP 0 cmH_2_O; PSV 0 cmH_2_O + PEEP 0 cmH_2_O or the T piece. All measurements were obtained after 15 minutes of each test. A 10-minute period of return to baseline state (with initial settings of ventilation parameters before the first weaning test) was performed between each test and before extubation. *WT* weaning test

A 15-minute period corresponding to a baseline state was first recorded (using PSV and PEEP set by the clinician in charge of the patient before inclusion). Patients were then randomly assessed using computer-driven software with five settings: PSV 7 and PEEP 7 cmH_2_O; PSV 0 and PEEP 7 cmH_2_O; PSV 7 and PEEP 0 cmH_2_O; PSV 0 and PEEP 0 cmH_2_O or the T piece. Each setting lasted 15 minutes with a 10-minute period of return to baseline steady state between each setting (Fig. 1). Steady state was defined clinically as a period sufficient to ensure clinical stability in respiratory and hemodynamic variables assessed by a physical exam which took into account heart rate, respiratory rate, paradoxical breathing pattern, accessory muscle use, grunting at end expiration and nasal flaring [17], and as previously performed by our group [18, 19].

After being explored with these five settings, and in the case of clinical success in the different weaning trials, patients were ventilated for 10 minutes using baseline state variables and then were extubated if the clinical state was judged adequate by the clinician in charge. A post-extubation measurement was performed 20 minutes after extubation using an oro-nasal oxygen mask with a flow of 5 L/minute (equivalent to inspired oxygen fraction (FiO_2_) of 0.4 [20]). According to our local protocol described in detail in a previous review [21], and after having achieved each step of the protocol, non-invasive ventilation was performed as a prophylactic routine measure in the immediate post-extubation period, for between 30 and 45 minutes every 4 to 6 h. Settings were adjusted to target the following: tidal volume (V_T_) 6–10 ml/kg of ideal body weight, respiratory rate (RR) 12–20 c/minute and pulse arterial oxygen saturation (SpO2) equal or above 95 %. Non-invasive ventilation was never performed before the end of the protocol.

### Measurements

All patients were studied in a semi-recumbent position with the head of the bed elevated to an angle from 30 to 45 degrees, according to patient comfort. [22] Procedures are detailed in the additional material. Briefly, the respiratory mechanics measurements comprised flow, airway pressure, esophageal (Pes) and gastric (Pga) pressure swings. Trans-diaphragmatic swings (Pdi) were calculated by subtracting Pes from Pga. Minute ventilation (VE), tidal volume (V_T_), inspiratory (Ti), expiratory time (Te), total cycle duration (Ttot) and RR were calculated from the numerical integration of the flow signal.

The inspiratory WOB per breath performed by the patient was calculated from a Campbell diagram taking into account the presence of intrinsic PEEP. Eesophageal and trans-diaphragmatic pressure-time products (PTPes and PTPdi) were also measured as previously reported [23, 24]. Analyses of arterial blood gases were obtained at the end of each test.

### Statistical analysis

All values are presented as mean ± SD. To assess differences between the weaning tests, we used the Friedman test and then pairwise comparisons with the Wilcoxon test if a significant difference appeared. Statistical analysis was performed by an independent statistician (NM) using R software© (R Foundation for Statistical Computing, Auckland, New Zealand).

Based on the literature review, we hypothesized that the post-extubation WOB would be similar to the T-tube WOB [25, 26] and would approximate 1.5 +/- 0.9 J/L in obese critically ill patients. We also hypothesized that WOB in PSV 7 cmH_2_O and PEEP 7 cmH_2_O would approximate 0.7 +/- 0.5 J/L [27]. Then, with an alpha risk at 0.05 and a power at 0.90, 12 patients would be needed. We decided to include 17 patients in order to make sure that 12 patients would complete the study. Significance was set at *p* < 0.01 after correction for the number of multiple comparisons, i.e., using the Bonferroni test.

## Results

### Patients

**Fig. 2:**
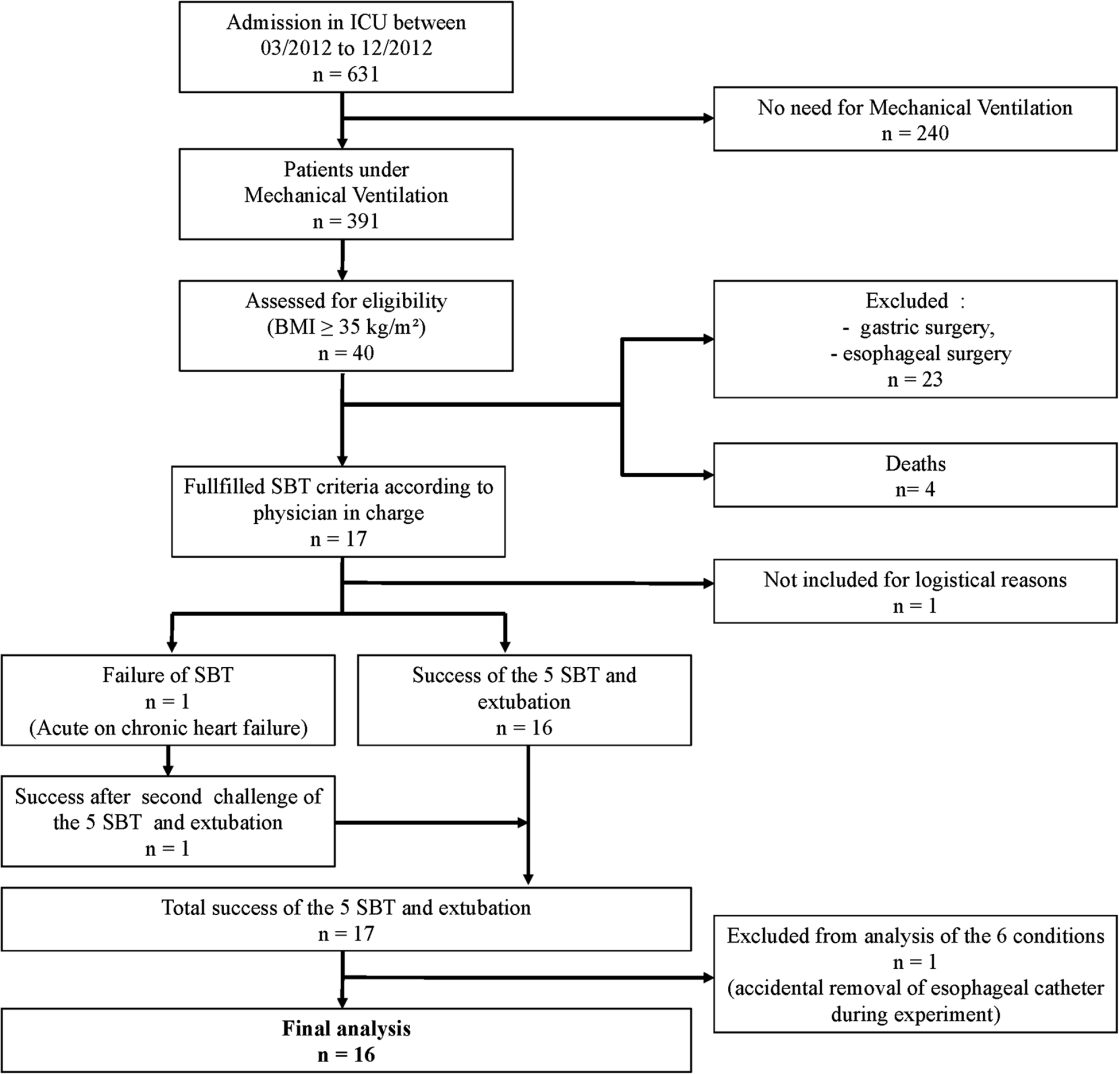
Flow chart of the study. One patient fulfilled the inclusion criteria but was not included because of extubation during the weekend with no investigator available. *BMI* body mass index, *SBT* spontaneous breathing trial

Between March and December 2012, 40 obese patients with body mass index ≥35 kg/m^2^ were admitted in our center. Among them, 17 met the inclusion criteria. Sixteen patients (13 women and 3 men) with mean body mass index of 44 kg/m^2^ (±8 kg/m^2^) were prospectively enrolled in the present study, as shown in Fig. 2. Characteristics of the subjects are detailed in Table 1. Mean duration of invasive mechanical ventilation before enrollment in the study was 6 days (±7 days). The five weaning tests were well-tolerated by all patients and all of them but one were successfully extubated.

**Table 1:** Characteristics of the patients

Patient	Sex	Age	SAPS II	Height	Weight	BMI	Underlying	Etiology of respiratory failure	ETT ID	MV before extubation	PSV at baseline	PEEP at baseline	Extubation failure	Outcome (D/S)
number		(years)		(cm)	(kg)	(kg/m^2^)	diseases		(mm)	(days)	(cmH_2_O)	(cmH_2_O)	(Y/N)	
1	F	83	109	150	80	35	CHF	Small bowel ischemia	7.5	7	8	6	N	D
2	F	85	68	163	115	43	NIDDM	Pneumonia	7.5	4	15	7	N	S
3	M	64	50	170	130	44	NIDDM	Acute pancreatitis	8	3	12	8	N	S
4	F	59	60	155	95	39	None	Peritonitis	7.5	3	12	8	N	S
5	F	49	66	160	174	67	COPD, OSA	Septic shock	7.5	6	10	10	N	S
6	F	25	29	172	145	49	None	Asthma	7.5	1	10	8	N	S
7	F	54	19	153	121	51	Asthma, HTN	Post abdominal surgery	7.5	1	10	8	N	S
8	M	37	54	180	130	40	None	Acute pancreatitis	8	14	8	10	N	S
9	F	78	90	155	87	36	None	Bowel obstruction	7.5	4	8	5	N	S
10	F	49	78	167	112	41	Asthma, OSA	Peritonitis	7.5	30	8	5	N	S
11	F	73	77	150	93	41	CHF, AF	Septic shock	7.5	4	12	6	N	D
12	F	50	45	162	94	36	None	Necrotizing fasciitis	7.5	2	9	7	N	S
13	M	63	64	175	180	56	NIDDM, HTN	Small bowel bleeding	7.5	1	8	10	N	S
14	F	43	48	155	105	43	OSA, home ventilation	Pneumonia	7.5	3	12	7	N	S
15	F	77	41	155	84	36	NIDDM, HTN	Pancreatitis	7.5	7	9	7	Y	S
16	F	50	64	164	124	46	OSA, home ventilation	Post abdominal surgery	7.5	8	14	8	N	S
Mean		59	60	162	117	44	.			6	10	7		
SD		17	22	9	30	8	.			7	2	2		

Abbreviations: *AF* atrial fibrillation, *BMI* body mass index; *CHF* chronic heart failure, *D* deceased; *ETT ID* endotracheal tube internal diameter; *F* female; *M* male; *HTN* hypertension, mechanical ventilation; *NIDDM* non-insulin-dependent diabetes mellitus; *OSA* obstructive sleep apnoea, *PEEP* positive end-expiratory pressure; *PSV* pressure support ventilation; *SAPS II* Simplified Acute Physiology Score II [34]; *S* survived

The first patient was initially unable to complete the five weaning tests. She was re-challenged 72 h later, and succeeded the tests and extubation. Seven days after extubation, she developed cardio-respiratory distress and was re-intubated. Patient number 15 developed hypoxemic acute respiratory failure and was re-intubated 12 h after extubation. One patient had accidental nasogastric catheter removal after extubation, preventing the measurement of respiratory muscle work variables after extubation. This patient was excluded from the final analysis.

### Respiratory variables and gas exchange

There was no statistical difference in any of the different respiratory variables (shown in Table 2) among the five weaning tests or at 20 minutes after extubation. In particular, differences in the RR/V_T_ ratio were not statistically significant between the five weaning tests or at 20 minutes after extubation. There was no statistically significant difference in arterial blood gases or hemodynamic variables among the six steps of the study, as shown in Table 3.

**Table 2:** Respiratory variables during the five different weaning tests and 20 minutes after extubation

	PSV	PSV	PSV	PSV	T piece	After extubation
	+7 cmH_2_O PEEP	0 cmH_2_O PEEP	+7 cmH_2_O PEEP	0 cmH_2_O PEEP		
	+7 cmH_2_O	+7 cmH_2_O	0 cmH_2_O	0 cmH_2_O		
Ti, s	0.90 ± 0.2	0.93 ± 0.23	0.82 ± 0.24	0.84 ± 0.28	0.81 ± 0.3	0.89 ± 0.43
Ttot, s	2.6 ± 0.8	2.4 ± 0.6	2.2 ± 0.6	2.1 ± 0.6	2.1 ± 0.6	2.2 ± 0.8
Ti/Ttot, %	35.7 ± 3.6	38.7 ± 4.2	37.8 ± 4.2	39.3 ± 4.4	38.7 ± 4.7	40.8 ± 4.3
V_T_, L	0.43 ± 0.12	0.41 ± 0.1	0.38 ± 0.1	0.37 ± 0.1	0.35 ± 0.1	0.36 ± 0.1
RR, breaths/minute	25 ± 6	26 ± 7	29 ± 6	30 ± 8	31 ± 7	30 ± 8
RR/V_T_, minutes/mL	64.5 ± 26.8	69.7 ± 25.0	83.1 ± 34.4	87.8 ± 36.4	94.7 ± 38.1	88.6 ± 34
VE, L/minute	10.3 ± 2.4	10.41 ± 2.9	10.8 ± 2.6	10.8 ± 3.3	10.5 ± 3.2	11.2 ± 4.4
PEEPi, cmH_2_O	1.1 ± 0.9	1.7 ± 1.2	2.5 ± 2.3	2.6 ± 2.2	2.4 ± 2.6	2.2 ± 2.3

There were no statistically significant differences between respiratory variables among the successive tests. Abbreviations: *PSV* pressure support ventilation; *PEEP* positive end-expiratory pressure; *PEEPi* intrinsic positive end-expiratory pressure; *RR* respiratory rate; *Ti* inspiratory time; *Ttot* total respiratory time; *VE* volume per minute; *V*_*T*_ tidal volume

**Table 3:** Arterial blood gases and hemodynamic variables during the five different weaning tests and at 20 minutes after extubation

	PSV	PSV	PSV	PSV	T piece	After extubation
	+7 cmH_2_O PEEP	0 cmH_2_O PEEP	+7 cmH_2_O PEEP	0 cmH_2_O PEEP		
	+7 cmH_2_O	+7 cmH_2_O	0 cmH_2_O	0 cmH_2_O		
Ph	7.45 ± 0.06	7.44 ± 0.06	7.44 ± 0.06	7.44 ± 0.06	7.43 ± 0.06	7.42 ± 0.06
Pa_CO2_, mmHg	41 ± 11	42 ± 11	43 ± 12	43 ± 12	44 ± 13	44 ± 10
Pa_O2_/FI_O2_	277 ± 76	257 ± 81	252 ± 73	230 ± 65	217 ± 65	224 ± 51
SBP, mmHg	148 ± 22	148 ± 26	148 ± 26	146 ± 30	150 ± 18	147 ± 24
DBP, mmHg	72 ± 12	71 ± 12	73 ± 12	72 ± 15	69 ± 13	70 ± 15
HR, beats/minute	96 ± 14	97 ± 16	98 ± 16	100 ± 16	99 ± 14	101 ± 15

There were no statistically significant differences between respiratory variables among the successive tests. Abbreviations: *DBP* diastolic blood pressure, *HR* heart rate, *ND* not done, *PEEP* positive end-expiratory pressure, *PSV* pressure support ventilation, *SBP* systolic blood pressure

### Inspiratory effort

**Fig. 3:**
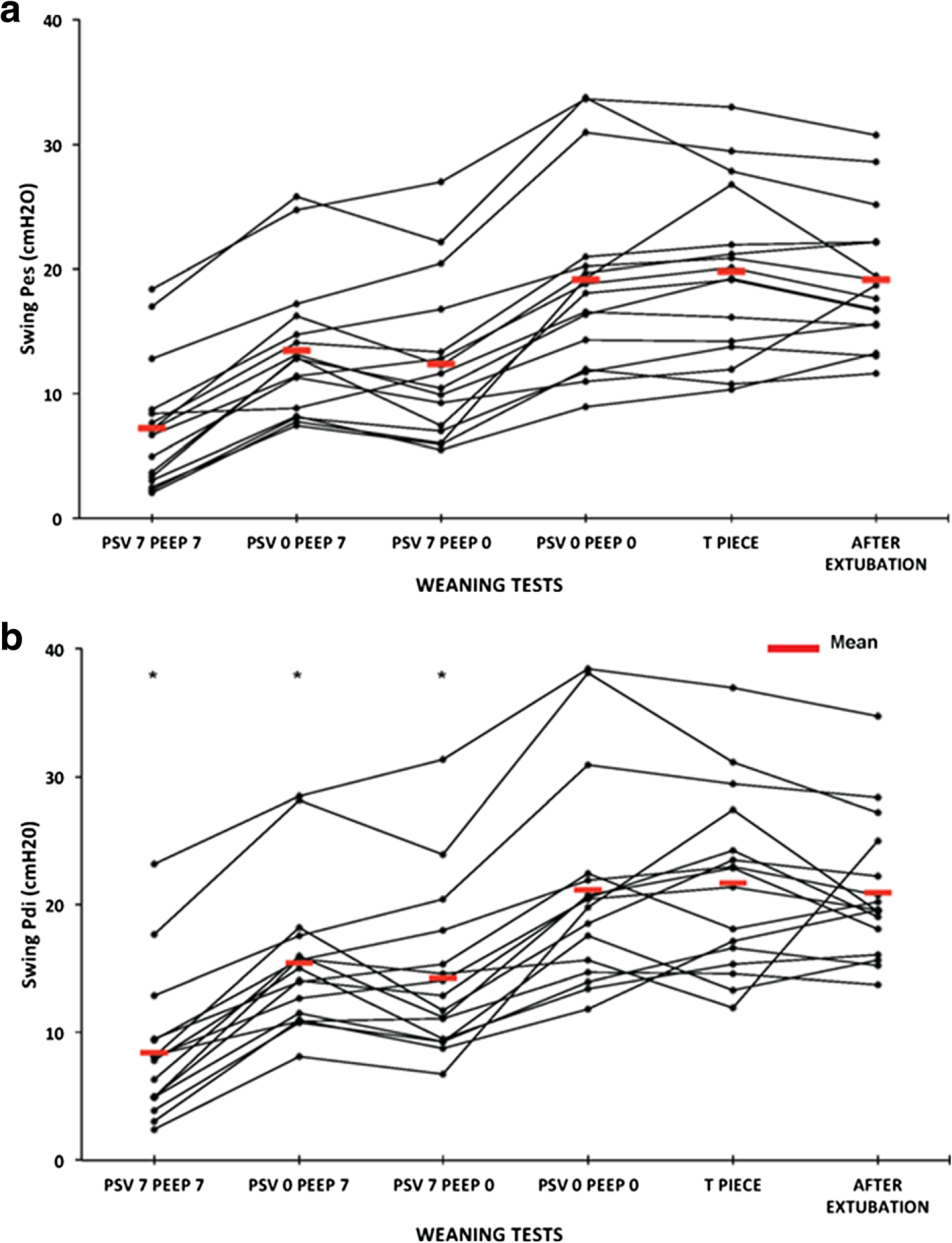
Esophageal (**a**) and trans-diaphragmatic (**b**) swings. Individual and mean changes in esophageal and trans-diaphragmatic swings during the five weaning tests and 20 minutes after extubation. All the tests show that the weaning tests that best reproduce respiratory muscle work after extubation were pressure support ventilation (*PSV*) 0 cmH_2_O + positive end-expiratory pressure (*PEEP*) 0 cmH_2_O and the T piece, with no statistically significant difference between the two. *p < 0.001 when compared with after extubation. *Pdi* transdiaphragmatic pressure, *pes* esophageal pressure

**Fig. 4:**
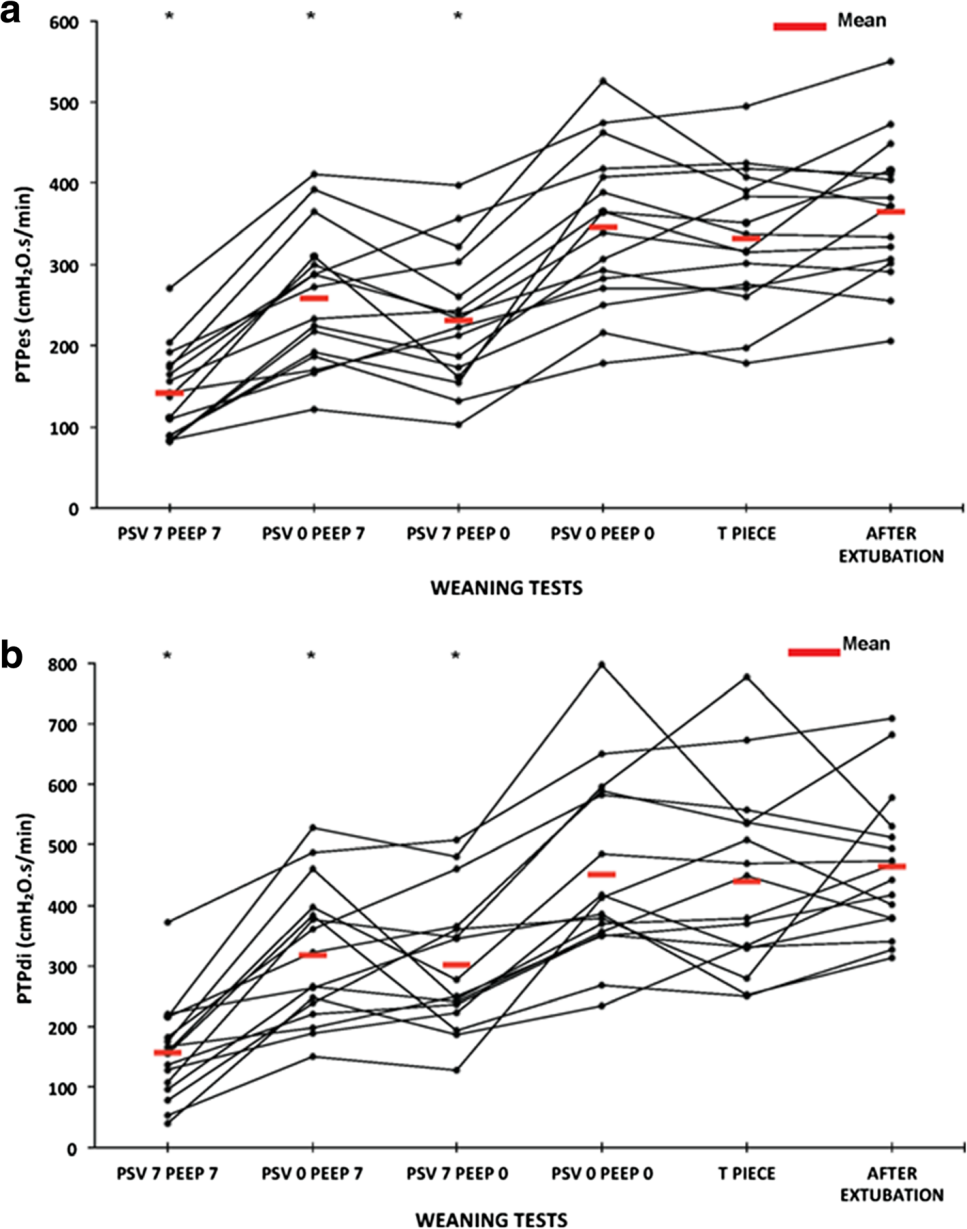
Esophageal (**a**) and trans-diaphragmatic (**b**) pressure time products. Individual and mean changes in esophageal and trans-diaphragmatic pressure time products during the five weaning tests and 20 minutes after extubation. All the tests show that the weaning tests that best reproduce respiratory muscle work after extubation were pressure support ventilation (*PSV*) 0 cmH_2_O+ positive end-expiratory pressure (*PEEP*) 0 cmH_2_O and the T piece, with no statistically significant difference between the two. *p < 0.001 when compared with after extubation. *PTPdi* trans-diaphragmatic pressure-time product, *PTPes* trans-esophageal pressure-time product

**Fig. 5:**
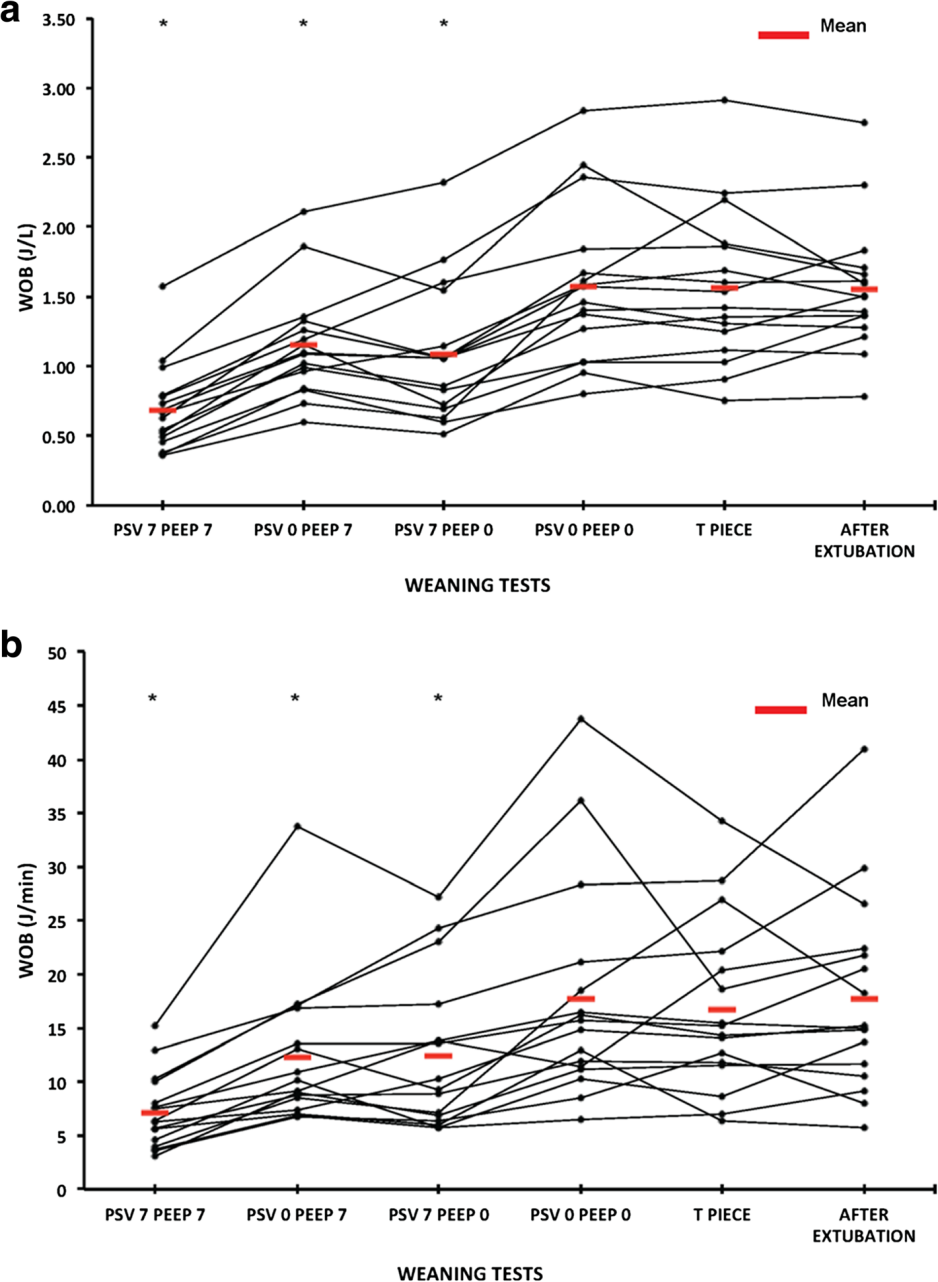
Work of breathing (WOB) in J/L (**a**) and in J/minute (**b**). Individual and mean changes in the WOB during the five weaning tests and 20 minutes after extubation. All the tests show that the weaning tests that best reproduced respiratory muscle work after extubation were pressure support ventilation (*PSV*) 0 cmH_2_O cmH_2_O + positive end-expiratory pressure (*PEEP*) 0 cmH_2_O and the T piece, with no statistically significant difference between the two. *p < 0.001 when compared with after extubation

**Fig. 6:**
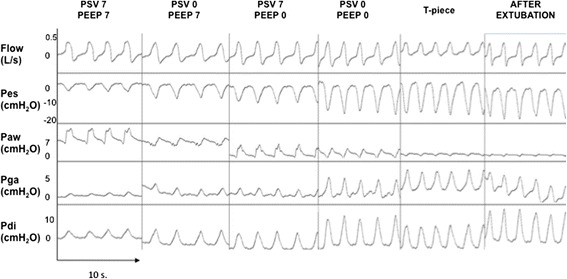
Ventilatory pattern during the five weaning tests and twenty minutes after extubation. One patient is presented with the acquisition of flow (L/s), esophageal (*Pes*, cmH_2_O), airway (*Paw*, cmH_2_O), gastric (*Pga*, cmH_2_O) and trans-diaphragmatic (*Pdi*, cmH_2_O) pressure signals. *PSV* pressure support ventilation, *PEEP* positive end-expiratory pressure

Figures 3, 4, and 5 show the individual and mean values of the main variables studied, and representative tracings of Pes, Pga and Pdi can be seen in Fig. 6. There was a significant difference in all respiratory effort variables (swings of Pes and Pdi, PTPes and PTPdi, WOB in J/L and in J/min) between the weaning tests and after the extubation period (*p* < 0.001) (Table 4). Weaning tests performed with positive pressure constantly overestimated post-extubation inspiratory effort. Inspiratory effort measured with either the T tube or PSV 0 + PEEP 0 cmH_2_O was not different to post-extubation inspiratory effort. We then identified both PSV 0 + PEEP 0 cmH_2_O and the T-piece trial as the weaning tests that reproduce post-extubation inspiratory effort and the WOB (Additional files 2, 3, 4, 5, 6 and 7).

**Table 4:** Inspiratory muscle effort during the five different weaning tests and 20 minutes after extubation

	PSV	PSV	PSV	PSV	T piece	After extubation
	+7 cmH_2_O PEEP	0 cmH_2_O PEEP	+7 cmH_2_O PEEP	0 cmH_2_O PEEP		
	+7 cmH_2_O	+7 cmH_2_O	0 cmH_2_O	0 cmH_2_O		
Swing Pes, cmH_2_O	7.2 ± 5.0*	13.4 ± 5.5*	12.3 ± 6.3*	19.1 ± 7.7	19.8 ± 7	19.1 ± 5.4
Swing Pdi, cmH_2_O	8.4 ± 5.5*	15.4 ± 5.7*	14.2 ± 6.4*	21.2 ± 8.1	21.7 ± 7.0	20.9 ± 5.5
PTP es, cmH_2_O.s/minute	141 ± 54*	259 ± 84*	231 ± 82*	346 ± 97	332.9 ± 85.9	365 ± 87
PTP di, cmH_2_O.s/minute	157 ± 80*	318 ± 113*	302 ± 111*	451 ± 151	439 ± 152	465 ± 117
WOB, J/L	0.69 ± 0.31*	1.15 ± 0.39*	1.09 ± 0.49*	1.58 ± 0.57	1.57 ± 0.56	1.56 ± 0.5
WOB, J/minute	7.15 ± 3.5*	12.2 ± 6.8*	12.4 ± 7.1*	17.7 ± 10.2	16.8 ± 8.0	17.8 ± 9.1

Abbreviations: *Pdi* trans-diaphragmatic pressure, *PEEP* positive end-expiratory pressure, *Pes* esophageal pressure, *PTPdi* trans-diaphragmatic pressure time product, *PTPes* esophageal pressure time product, *PSV* pressure support ventilation, *WOB* work of breathing. *p < 0.001 when compared with after extubation

## Discussion

To our knowledge, this is the first physiological study that specifically investigates the inspiratory effort during weaning of mechanical ventilation in a population of critically ill morbidly obese patients. The main result of this study is that for obese patients, the T piece and PSV 0 + PEEP 0 cmH_2_O weaning tests are the tests that best predict post-extubation inspiratory effort and WOB.

Because of a lack of consensus on the best test to use before extubation in this population, we aimed to determine which one reflects the breathing effort after extubation. Some authors described extubation of obese patients after a 30-minute period of CPAP 5 cmH_2_O [14], others after a trial of FiO_2_ 100 % combined with a CPAP of 10 cmH_2_O. [15] An ongoing multicenter observational study in France (FREEREA study), will provide some epidemiological data about weaning and extubation in this particular population. The preliminary results (unpublished) show that among 64 critically ill morbidly obese patients extubated, 22 (34 %) were extubated after a T tube, 28 (44 %) after a low PSV trial, 12 (19 %) with no spontaneous breathing trial and 2 (3 %) after a different weaning trial. These data justify our study as there is wide heterogeneity of extubation practice in this population, with a high proportion of patients being extubated from a substantial level of support.

Our study presents limitations. First, we investigated the inspiratory effort indexes twenty minutes after extubation and the study was not designed to explore long-term consequences of several weaning tests on oxygenation, end-expiratory lung volume or outcome. Because outcome was not a study endpoint, we cannot make any final recommendation about which weaning test is associated with the highest rate of weaning success. Ideally, a weaning test would perfectly predict the ability of the patient to breathe alone and without being ventilatory assisted by simulating the post-extubation respiratory constraint [26]. Second, post-extubation intermittent non-invasive ventilation is routinely used in our unit for high-risk patients [14, 21] to rest the inspiratory muscles and improve lung aeration. It may have contributed to our low rate of re-intubation (6 %).

The present study focused on morbidly obese patients and found results consistent with the studies published by Straus et al. [25] and Cabello et al. [8], which included non-obese patients. We report that the T piece and PSV 0 + PEEP 0 cmH_2_O weaning tests were the two tests that best approximated the WOB after extubation. We also found that the PSV 7 + PEEP 0 cmH_2_O test leads to a major underestimation of the WOB after extubation in obese patients with significantly less inspiratory effort in comparison with both the T piece test and 20 minutes after extubation. Straus et al demonstrated that post-extubation WOB was well-approximated by the WOB during a T-piece test and that the endotracheal tube was responsible for about 11 % of the total work of breathing. [25] More recently, Cabello et al. compared a spontaneous breathing trial on a T-piece with low PSV (7 cm H_2_O) with or without PEEP in a subpopulation of patients with heart failure who were difficult to wean. [8] The authors concluded that performing the weaning test while maintaining a positive pressure in the circuit underestimates the post-extubation WOB and unmasks a possible effect on left ventricular function, and suggested the T piece as the weaning test of choice in these patients.

In a landmark physiological study, Brochard et al. demonstrated that breathing through the T piece overestimates the WOB by 27 ± 18 % compared to the post-extubation period [26]. Contrary to the present study, Brochard et al. included a high proportion of patients with chronic obstructive pulmonary disease, and used ventilators with higher ventilatory circuit-resistive load [28] and lower pressurization performance, especially in terms of inspiratory-trigger-imposed WOB [29, 30].

As compared to the literature on non-obese patients, WOB values evaluated in the present study were higher [26, 31]. In morbidly obese patients, an elevation of pharyngeal collapsibility and upper airway resistance related to fatty deposits on pharynx and oral soft tissue and associated with local inflammation can increase the WOB [32]. Weaning trials performed with positive pressure underestimated post-extubation WOB by 33 % (0.5 J/L) up to 50 % (0.8 J/L) according to the ventilator setting. An increase of 0.5–0.8 J/L represents a significant additional workload, as WOB in healthy subjects during quiet breathing is about 0.35–0.5 J/L [33, 34]. Furthermore, WOB ≥0.8 J/L has been reported as being associated with weaning failure [35]. Extubating an obese patient after having performed a weaning test without positive pressure could lead to early onset atelectasis if the patient was unable to control for end-expiratory lung volume without PEEP.

## Conclusions

For the first time the present study reports new insights into respiratory physiology in morbidly obese critically ill candidates to be weaned from the ventilator. These data may be useful for clinicians managing these challenging patients and help make difficult decisions about extubation. We report that either a T piece or a PSV 0 and PEEP 0 cmH_2_O test are the trials that predict post-extubation work of breathing in morbidly obese patients. The consequences on mid-term oxygenation and lung aeration, and on the weaning success rate of such weaning tests were, however, not studied.

## Additional files

Additional file 1: Supplementary material. (DOCX 44 kb)

Additional file 2: **Figure S1.** difference in esophageal pressure between each test and the post-extubation period. *Dashed line* represents the absence of difference between the test and the post-extubation period. (JPG 44 kb)

Additional file 3: **Figure S2.** difference in the trans-diaphragmatic pressure between each test and the post-extubation period. *Dashed line* represents the absence of difference between the test and the post-extubation period. (JPG 48 kb)

Additional file 4: **Figure S3.** difference in the esophageal pressure time product between each test and the post-extubation period. *Dashed line* represents the absence of difference between the test and the post-extubation period. (JPG 49 kb)

Additional file 5: **Figure S4.** difference in the trans-diaphragmatic pressure time product between each test and the post-extubation period. *Dashed line* represents the absence of difference between the test and the post-extubation period. (JPG 47 kb)

Additional file 6: **Figure S5.** difference in the work of breathing expressed in J/l between each test and the post-extubation period. *Dashed line* represents the absence of difference between the test and the post-extubation period. (JPG 44 kb)

Additional file 7: **Figure S6.** difference in the work of breathing expressed in J/min between each test and the post-extubation period. Dashed line represents the absence of difference between the test and the post-extubation period. (JPG 44 kb)

## Abbreviations

BMI: Body mass index
CPAP: Continuous positive airway pressure
FiO_2_: Inspired oxygen fraction
ICU: Intensive Care Unit
Pdi: Trans-diaphragmatic pressure
PEEP: Positive end-expiration pressure
Pes: Esophageal pressure
Pga: Gastric pressure
PSV: Pressure support ventilation
PTPdi: Trans-diaphragmatic pressure-time product
PTPes: Trans-esophageal pressure-time product
RR: Respiratory rate
SBT: Spontaneous breathing trial
SpO_2_: Pulse arterial oxygen saturation
Te: Expiratory time
Ti: Inspiratory time
Ttot: Total cycle duration
VE: Minute ventilation
V_T_: Tidal volume
WOB: Work of breathing
