# Supplementary material for: Spontaneous breathing trial and post-extubation work of breathing in morbidly obese critically ill patients
Source: Crit Care. 2016 Oct 27;20:346. doi: 10.1186/s13054-016-1457-4 (PMC5081985; doi:10.1186/s13054-016-1457-4)
Supplement: Supplementary file 1 — Supplementary material. (DOCX 44 kb) [file 13054_2016_1457_MOESM1_ESM.docx]

**Supplementary material**

**Spontaneous breathing trial and post-extubation work of breathing in morbidly obese critically ill patients**

Martin Mahul ^1*^; Boris Jung^1,4*^; Fabrice Galia ^1^; Nicolas Molinari ^2^; Audrey de Jong ^1^; Yannaël Coisel ^1,4^; Rosanna Vaschetto ^3^, Stefan Matecki ^4^; Gérald Chanques ^1,4^; Laurent Brochard ^5^; Samir Jaber ^1,4^

* These authors contributed equally to this work.

**Authors affiliations:**

^1^ Intensive Care Unit, Anaesthesia and Critical Care Department, Saint Eloi Teaching Hospital, Centre Hospitalier Universitaire Montpellier, France

^2^ Department of Statistics, University of Montpellier Lapeyronie Hospital, UMR 729 MISTEA, Montpellier, France

^3^ Anaesthesia and Intensive Care Medicine, Maggiore della Carità Hospital, Novara, Italy

^4^ Centre National de la Recherche Scientifique (CNRS 9214) - Institut National de la Santé et de la Recherche Médicale (INSERM U-1046), Montpellier University, Montpellier, France

^5^ Keenan Research Centre, St Michael’s Hospital, Toronto; Interdepartmental Division of Critical Care Medicine, University of Toronto, Canada

* These authors contributed equally to this work.

**Correspondence** to Prof. Jaber, Department of Critical Care Medicine and Anesthesiology, Saint Eloi Teaching Hospital; 80 avenue Augustin Fliche, F-34295 Montpellier, Cedex 5 and Institut National de la Santé et de la Recherche Médicale / Centre National de la Recherche Scientifique (INSERM U-1046, CNRS 9234), Montpellier University, Montpellier, France

Phone: (33) 4 67 33 72 71

Fax: (33) 4 67 33 74 48

E-mail: [s-jaber@chu-montpellier.fr](mailto:s-jaber@chu-montpellier.fr)

Email adresses :

Martin Mahul : m-mahul@chu-montpellier.fr

Boris Jung : b-jung@chu-montpellier.fr

Fabrice Galia : f-galia@chu-montpellier.fr

Nicolas Molinari : n-molinari@chu-montpellier.fr

Audrey de Jong : a-de_jong@chu-montpellier.fr

Yannaël Coisel : yannacoi@free.fr

Rosanna Vaschetto : rosanna.vaschetto@med.unipmn.it

Stefan Matecki : s-matecki@chu-montpellier.fr

Gérald Chanques : g-chanques@chu-montpellier.fr

Laurent Brochard : BrochardL@smh.ca

Samir Jaber : s-jaber@chu-montpellier.fr

Methods

Patients

Patients with the following respiratory parameters: FiO_2_ $\leq$ 50% with PEEP ≤ 10 cmH_2_O were screened daily for enrolment in the study. Contraindications to insertion of an esophageal catheter were upper gastrointestinal bleeding, recent esophageal or gastric surgery or history of esophageal perforation. We did not considered as eligible patients with high risk of weaning test failure [1]: PaO_2_/FiO_2_< 150, pressure support ventilation (PSV) superior to 20 cmH_2_O, PEEP > 10 cmH_2_O, mean arterial blood pressure < 70 mmHg or need for vasopressive drugs. Pregnant women and children < 18 years old were also not considered eligible.

Experimental Procedure and Study Design

According to our local protocol, we stopped enteral nutrition 2h before starting the weaning tests. Before starting the weaning test protocol, a 15 minutes base-line period was respected in which the patient was ventilated and stabilized with the ventilator settings he had before the protocol. Because of immediate post-extubation cough and incomfort that would have influenced the respiratory pattern, a 20 minutes delay was set before measuring the post-extubation variables.

Inspired oxygen concentration was kept constant during the study period and all patients were blinded to the changes of the ventilator settings, except for the base-line return. Respiratory tract suctioning was done only if necessary, preferably during the base-line state periods.

Decision to extubate or reinstitute mechanical ventilation was made by the primary physician, blinded to the results of the different weaning tests, and based upon the failure criteria of weaning test as defined by the European guidelines.[1]

Monitoring of ventilatory parameters.

Flow and tidal volume (VT) were measured between the endotracheal tube and the Y piece of the ventilator circuit with a Fleisch n°1 pneumotachograph (Fleisch, Lausanne, Switzerland). Airway pressure was measured via a pressure transducer (MP45 Validyne, Northridge, CA) through the pneumotachograph. Measurement of esophageal and gastric swing pressures (Inspiratory minus expiratory pressures) were performed with a double balloon catheter (Nutrivent®, SIDAM, Italy). This catheter was inserted through the nose after the application of topical anesthesia. Each balloon was inflated with 4 mL of air and was connected to a differential pressure transducer. Proper positioning of esophageal balloon (lower third portion of esophagus) was verified by an occlusion test previously described by Baydur et al [2] while proper positioning of gastric balloon was verified by application of repeated manual gastric pressure on the patient's abdomen by the physician in charge to observe fluctuations in gastric pressure. All pressure signals were recorded through pressure transducer (MP45 Validyne, Northridge, CA) and were sampled and digitized at 200 Hz using an analog-to-digital converter system (MP35; Biopac Systems, Santa Barbara, CA). Changes in pleural and abdominal pressures were estimated respectively from variations in esophageal (Pes) and gastric (Pga) pressures during inspiratory and expiratory time (Swing Pes and Swing Pgas, expressed in cm H_2_O). Trans-diaphragmatic swing (Pdi) was calculated by subtracting Pes from Pga. Minute ventilation (VE), VT, inspiratory (Ti) and expiratory time (Te), total cycle duration (Ttot), respiratory rate (RR), were calculated from the numerical integration of the flow signal. Pes values at zero-flow were considered as beginning and end of inspiration and we considered that any difference between initial Pes and the zero-flow point indicates intrinsic PEEP. [3] The intrinsic PEEP was corrected for expiratory muscle activity, as detected on Pga tracings. [4] Expired tidal volume was used for data analysis. We measured the ratio of expired to inspired tidal volume and applied a correction factor equal to this ratio to the flow signal as previously described. [5]

Inspiratory effort

The inspiratory work of breathing per breath performed by the patient was calculated from a Campbell diagram by integrating the area between the inspiratory esophageal pressure, the inspiratory VT curve and the static Pes-volume curve of the chest wall, using a theoretical value for the chest wall compliance (4% of the predicted value for the vital capacity per cm of water). [6] Although the use of this theoretical value may result in some error, this was expected to be identical for all periods and not affecting the validity of comparisons. Ten to fifteen consecutives constant breathing cycles minimum (e.g "no caught, no shallow breathing, no maximal inspiratory effort, no esophageal spasm, no artifacts) were recorded during the 3 last minutes of each SBT. Even if the use of this theoretical value may result in approximation, this was expected to be identical for all periods and not to affect the validity of comparisons, as previously described. [6,7] Inspiratory WOB was expressed as the work per volume unit (joules per liter) or as the work per time unit (joules per minute). Esophageal and trans-diaphragmatic pressure-time products (PTPes and PTPdi) were also measured as reported elsewhere. [8,9]. PTPes was calculated as the integral of pressure during the time of inspiratory muscular effort. In the same way, the PTPdi was calculated by measuring the area under the Pdi signal from the onset of its positive increasing to its final deflection at the end of inspiratory muscular effort. The average PTPes and PTPdi were multiplied by the respiratory rate to obtain the PTPdi/min (cm H_2_O.s/min-1).

Standard Monitoring.

Standard three-lead monitoring electrodes continuously recorded heart rate and rhythm. Oxygen saturation was continuously monitored using pulse oxymetry. Systolic and diastolic arterial blood pressures were continuously monitored through a 20-gauge catheter inserted in a radial or femoral artery. Arterial Blood Gases analysis (GEM Premier 3000 analyzer; Instrumentation Laboratory, Lexington, MA) were obtained at the end of each weaning test and before EELV oxygen wash-in/wash-out measurement.

Statistical Analysis

All values are presented as mean ± SD. To assess differences between the weaning tests, we used the Friedman test and then pairwise comparisons with Wilcoxon test if a significant difference appeared. Statistical analysis was performed by an independent statistician (NM) using R software © (R Foundation for Statistical Computing, Auckland, New Zealand).

Based on the literature review, we hypothesized that the post extubation WOB would be similar than the T-Tube WOB [7,10] and would approximate 1.5 +/- 0.9 J/L in obese critically ill patients. We also hypothesized that WOB in PSV 7 cmH_2_O and PEEP 7 cmH_2_O would approximate 0.7 +/- 0.5 J/L [11]. Then, with an alpha risk at 0.05 and a power at 0.90, twelve patients would be needed. We decided to include 17 patients in order to make sure that 12 patients will end the study. Significance was set at p < 0.01 after correction for the number of multiple comparisons, i.e. 5, by Bonferroni test.

Results

Three patients (Patient #2, #3 and #8), not previously diagnosed with obstructive lung disease, did show a clinically relevant intrinsic PEEP as reported in the table S2. The need to compensate for lung hyperinflation during inspiration probably explains higher trends for WOB, oesophageal and diaphragmatic swings during the 2 weaning trials without pressure support (PSV 0 PEEP 0 and T piece) and during the post extubation measure.

Table S1: Randomization table.

| Patient N° | WEANING TEST 1 | WEANING TEST 2 | WEANING TEST 3 | WEANING TEST 4 | WEANING TEST 5 |
| --- | --- | --- | --- | --- | --- |
| 1 | PS 7 PEEP 7 | PS 0 PEEP 7 | PS 7 PEEP 0 | PS 0 PEEP 0 | TT |
| 2 | PS 7 PEEP 7 | PS 0 PEEP 0 | TT | PS 7 PEEP 0 | PS 0 PEEP 7 |
| 3 | PS 0 PEEP 7 | PS 7 PEEP 0 | PS 0 PEEP 0 | PS 7 PEEP 7 | TT |
| 4 | PS 0 PEEP 0 | PS 7 PEEP 0 | PS 7 PEEP 7 | TT | PS 0 PEEP 7 |
| 5 | TT | PS 7 PEEP 7 | PS 0 PEEP 0 | PS 0 PEEP 7 | PS 7 PEEP 0 |
| 6 | PS 0 PEEP 7 | PS 0 PEEP 0 | TT | PS 7 PEEP 0 | PS 7 PEEP 7 |
| 7 | PS 7 PEEP 0 | TT | PS 0 PEEP 7 | PS 0 PEEP 0 | PS 7 PEEP 7 |
| 8 | PS7PEEP7 | PS0PEEP7 | PS7PEEP0 | PS0PEEP0 | TT |
| 9 | PS7PEEP0 | PS0PEEP0 | PS7PEEP7 | PS0PEEP7 | TT |
| 10 | PS7PEEP0 | PS0PEEP0 | TT | PS7PEEP7 | PS0PEEP7 |
| 11 | PS7PEEP7 | PS0PEEP7 | PS7PEEP0 | TT | PS0PEEP0 |
| 12 | PS 7 PEEP 7 | PS 0 PEEP 7 | PS 7 PEEP 0 | PS 0 PEEP 0 | TT |
| 13 | TT | PS 7 PEEP 0 | PS 0 PEEP 7 | PS 0 PEEP 0 | PS 7 PEEP 7 |
| 14 | PS 7 PEEP 0 | TT | PS 0 PEEP 7 | PS 7 PEEP 7 | PS 0 PEEP 0 |
| 15 | TT | PS7 PEEP 0 | PS 0 PEEP 7 | PS 7 PEEP 7 | PS 0 PEEP 0 |
| 16 | PS 0 PEEP 0 | TT | PS 7 PEEP 0 | PS 7 PEEP 7 | PS 0 PEEP 7 |

PEEP: End Expiratory Positive Pressure; PS: Pressure Support; TT: T piece

Table S2: Individual intrinsic PEEP (cmH_2_O) for the 16 patients during the 5 weaning tests and post extubation. In bold are presented the PEEPi of these 3 patients.

| Patient | AI7 PEP7 | AI 0 PEP 7 | AI 7 PEP 0 | AI 0 PEP 0 | T-piece | Post Extubation |
| --- | --- | --- | --- | --- | --- | --- |
| 1 | 1.1 | 1.6 | 1.4 | 1.2 | 0.2 | 0.3 |
| 2 | 3.6 | 4.3 | 8.4 | 8.3 | 9.3 | 7.1 |
| 3 | 2.08 | 2.7 | 5.9 | 4.3 | 3.6 | 6.3 |
| 4 | 1 | 1.7 | 2.5 | 3 | 2 | 2 |
| 5 | 1.3 | 1.2 | 3 | 3.3 | 4 | 3 |
| 6 | 0.4 | 0.9 | 0.6 | 1.3 | 0.9 | 0.7 |
| 7 | 0 | 0.2 | 0.8 | 1 | 0.1 | 0.3 |
| 8 | 0.6 | 2.6 | 3.3 | 3.7 | 1.4 | 3.1 |
| 9 | 0.5 | 0.7 | 0.5 | 0.6 | 0.5 | 0.8 |
| 10 | 1 | 0.8 | 1.50 | 1.3 | 1 | 2 |
| 11 | 0.6 | 1.5 | 0.9 | 1.4 | 1.3 | 1.1 |
| 12 | 0.4 | 0.3 | 0.2 | 0 | 0.2 | 0 |
| 13 | 1.1 | 1.9 | 1.7 | 1.5 | 0.2 | 0.2 |
| 14 | 0.9 | 1.1 | 1.5 | 0.9 | 2.1 | 2.3 |
| 15 | 0.9 | 1.4 | 2.2 | 4.5 | 6.7 | 5.8 |
| 16 | 1.7 | 4 | 5.1 | 5.1 | 4.8 | 1 |

REFERENCES

1. Boles JM, Bion J, Connors A, Herridge M, Marsh B, Melot C, et al. Weaning from mechanical ventilation. European Respiratory Journal. 2007;29:1033–56.

2. Baydur A, Behrakis PK, Zin WA, Jaeger M, Milic-Emili J. A simple method for assessing the validity of the esophageal balloon technique. Am. Rev. Respir. Dis. 1982;126:788–91.

3. Mancebo J, Albaladejo P, Touchard D, Bak E, Subirana M, Lemaire F, et al. Airway occlusion pressure to titrate positive end-expiratory pressure in patients with dynamic hyperinflation. Anesthesiology. 2000;93:81–90.

4. Lessard MR, Lofaso F, Brochard L. Expiratory muscle activity increases intrinsic positive end-expiratory pressure independently of dynamic hyperinflation in mechanically ventilated patients. Am. J. Respir. Crit. Care Med. 1995;151:562–9.

5. Vitacca M, Ambrosino N, Clini E, Porta R, Rampulla C, Lanini B, et al. Physiological response to pressure support ventilation delivered before and after extubation in patients not capable of totally spontaneous autonomous breathing. Am. J. Respir. Crit. Care Med. 2001;164:638–41.

6. Jaber S, Sebbane M, Verzilli D, Matecki S, Wysocki M, Eledjam J-J, et al. Adaptive support and pressure support ventilation behavior in response to increased ventilatory demand. Anesthesiology. 2009;110:620–7.

7. Brochard L, Rua F, Lorino H, Lemaire F, Harf A. Inspiratory pressure support compensates for the additional work of breathing caused by the endotracheal tube. Anesthesiology. 1991;75:739–45.

8. Sassoon CS, Light RW, Lodia R, Sieck GC, Mahutte CK. Pressure-time product during continuous positive airway pressure, pressure support ventilation, and T-piece during weaning from mechanical ventilation. Am. Rev. Respir. Dis. 1991;143:469–75.

9. Jaber S, Carlucci A, Boussarsar M, Fodil R, Pigeot J, Maggiore S, et al. Helium-oxygen in the postextubation period decreases inspiratory effort. Am. J. Respir. Crit. Care Med. 2001;164:633–7.

10. Straus C, Louis B, Isabey D, Lemaire F, Harf A, Brochard L. Contribution of the endotracheal tube and the upper airway to breathing workload. Am. J. Respir. Crit. Care Med. 1998;157:23–30.

11. Mehta S, Nelson DL, Klinger JR, Buczko GB, Levy MM. Prediction of post-extubation work of breathing. Crit. Care Med. 2000;28:1341–6.
