# Supplementary figures and images for: Spontaneous breathing trial and post-extubation work of breathing in morbidly obese critically ill patients
Source: Crit Care. 2016 Oct 27;20:346. doi: 10.1186/s13054-016-1457-4 (PMC5081985; doi:10.1186/s13054-016-1457-4)

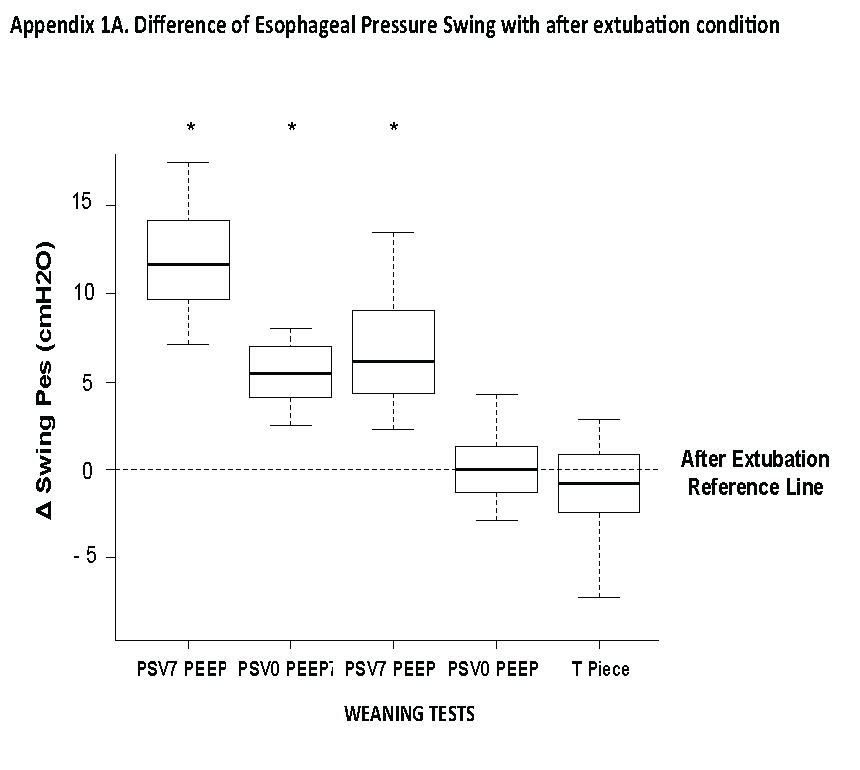

Supplement: Supplementary file 2 — Figure S1. difference in esophageal pressure between each test and the post-extubation period. Dashed line represents the absence of difference between the test and the post-extubation period. (JPG 44 kb) [file 13054_2016_1457_MOESM2_ESM.jpg]

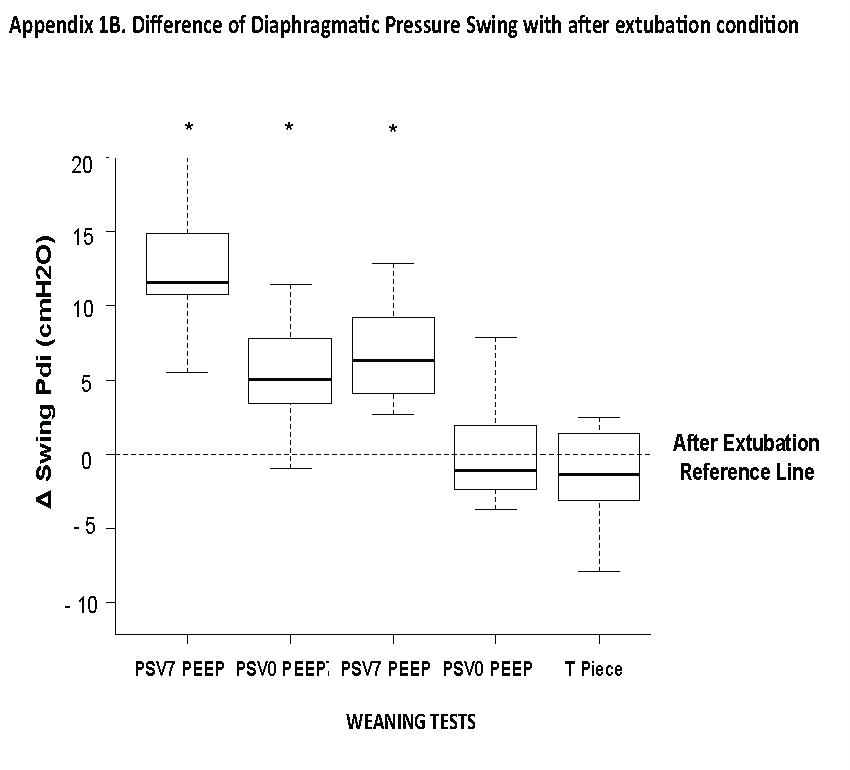

Supplement: Supplementary file 3 — Figure S2. difference in the trans-diaphragmatic pressure between each test and the post-extubation period. Dashed line represents the absence of difference between the test and the post-extubation period. (JPG 48 kb) [file 13054_2016_1457_MOESM3_ESM.jpg]

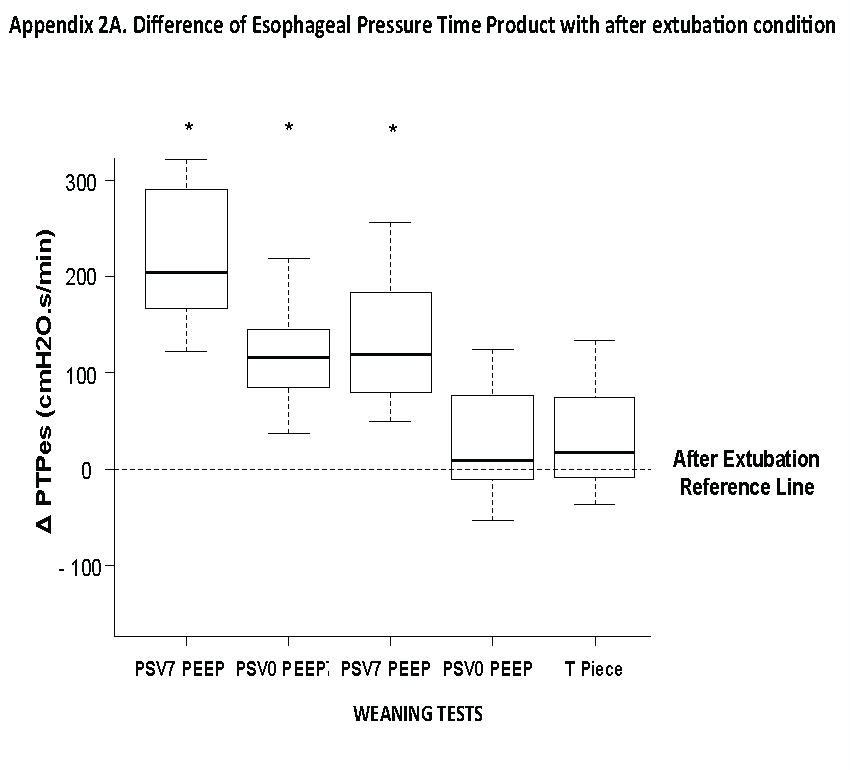

Supplement: Supplementary file 4 — Figure S3. difference in the esophageal pressure time product between each test and the post-extubation period. Dashed line represents the absence of difference between the test and the post-extubation period. (JPG 49 kb) [file 13054_2016_1457_MOESM4_ESM.jpg]

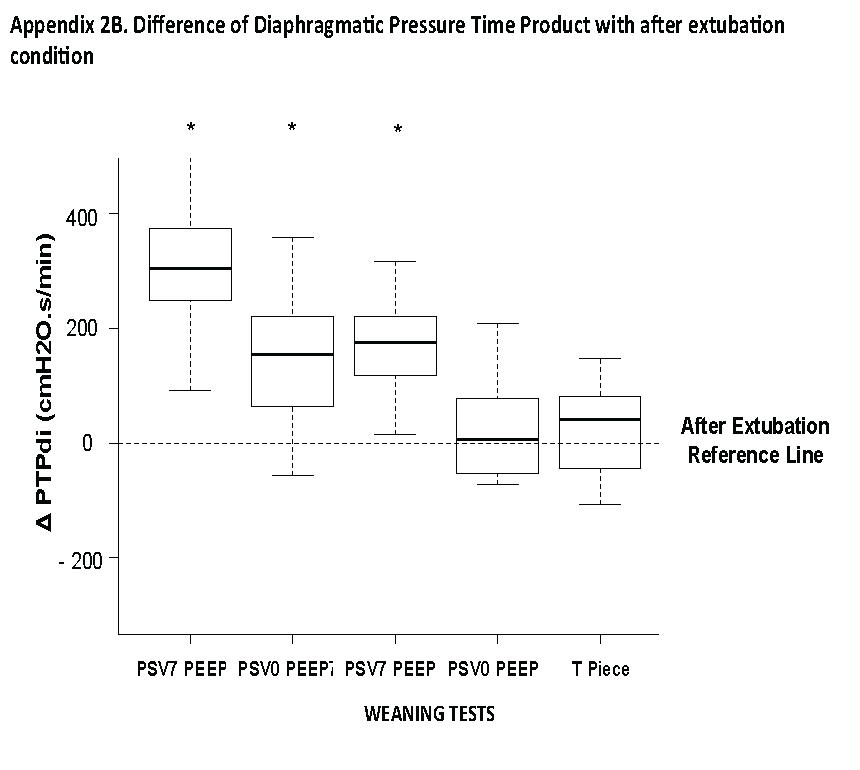

Supplement: Supplementary file 5 — Figure S4. difference in the trans-diaphragmatic pressure time product between each test and the post-extubation period. Dashed line represents the absence of difference between the test and the post-extubation period. (JPG 47 kb) [file 13054_2016_1457_MOESM5_ESM.jpg]

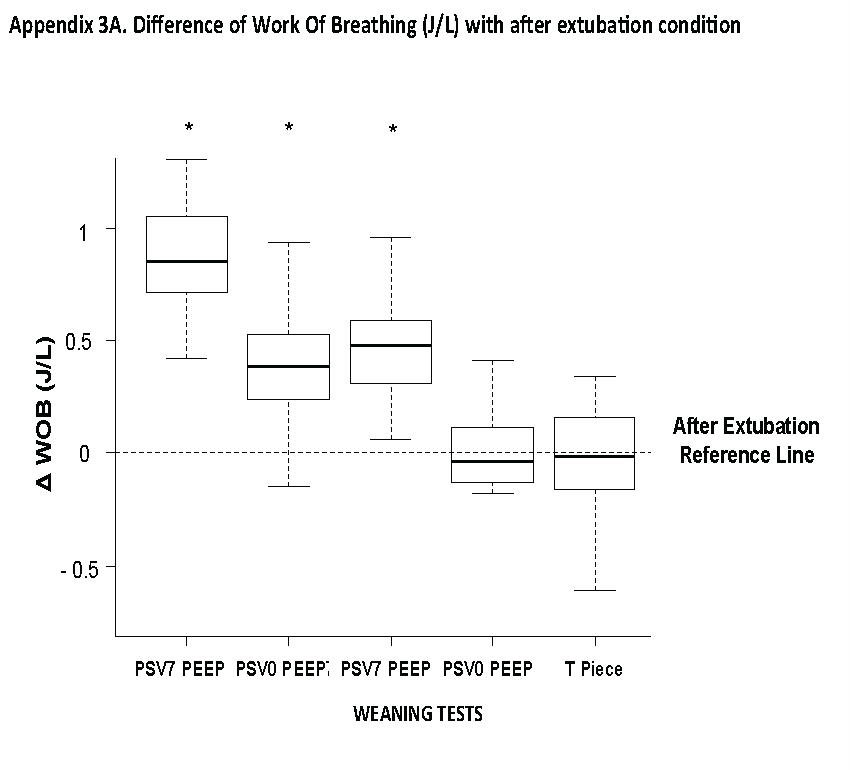

Supplement: Supplementary file 6 — Figure S5. difference in the work of breathing expressed in J/l between each test and the post-extubation period. Dashed line represents the absence of difference between the test and the post-extubation period. (JPG 44 kb) [file 13054_2016_1457_MOESM6_ESM.jpg]

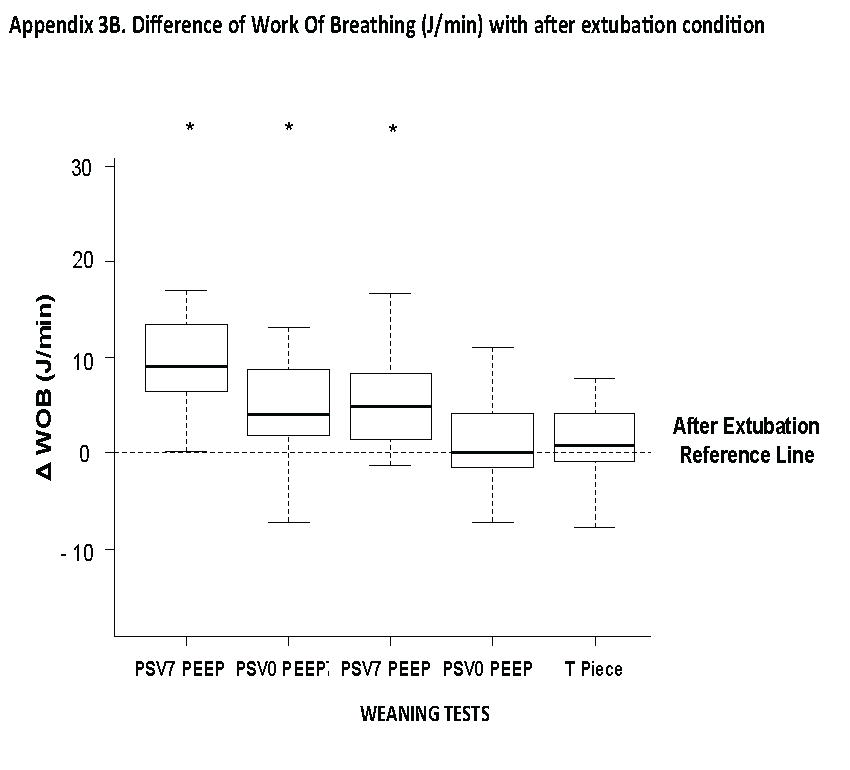

Supplement: Supplementary file 7 — Figure S6. difference in the work of breathing expressed in J/min between each test and the post-extubation period. Dashed line represents the absence of difference between the test and the post-extubation period. (JPG 44 kb) [file 13054_2016_1457_MOESM7_ESM.jpg]
